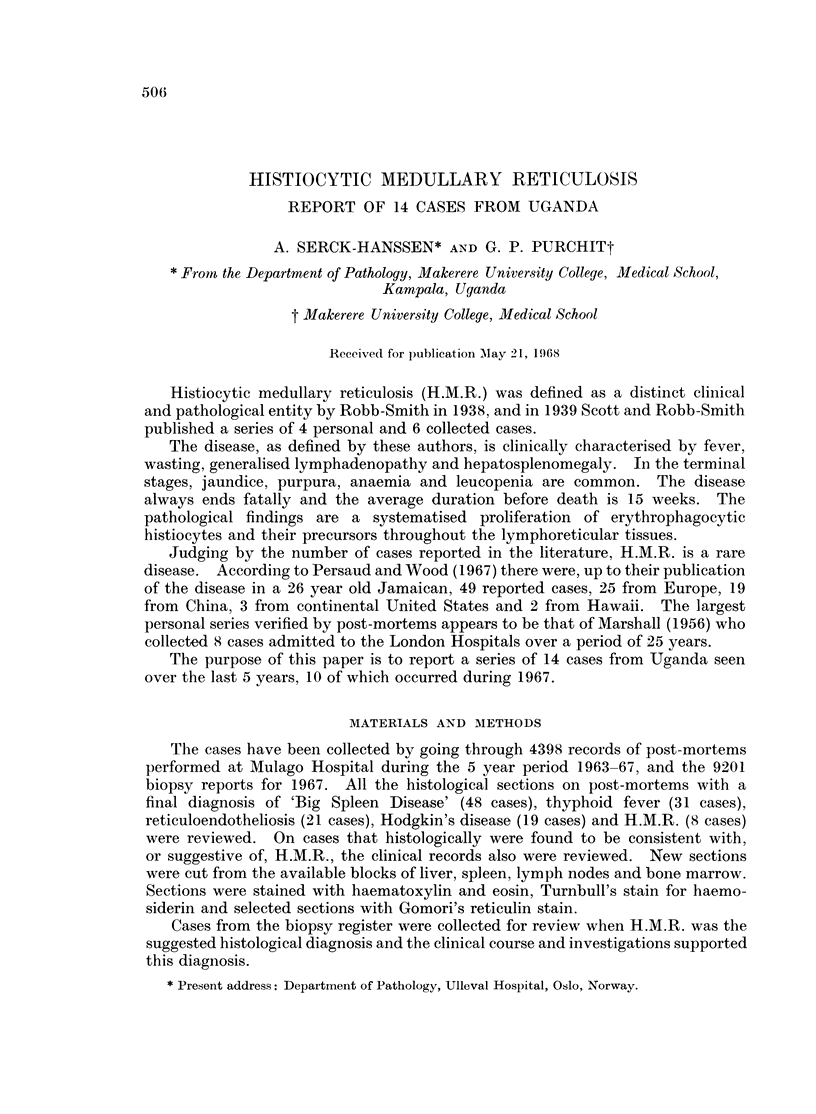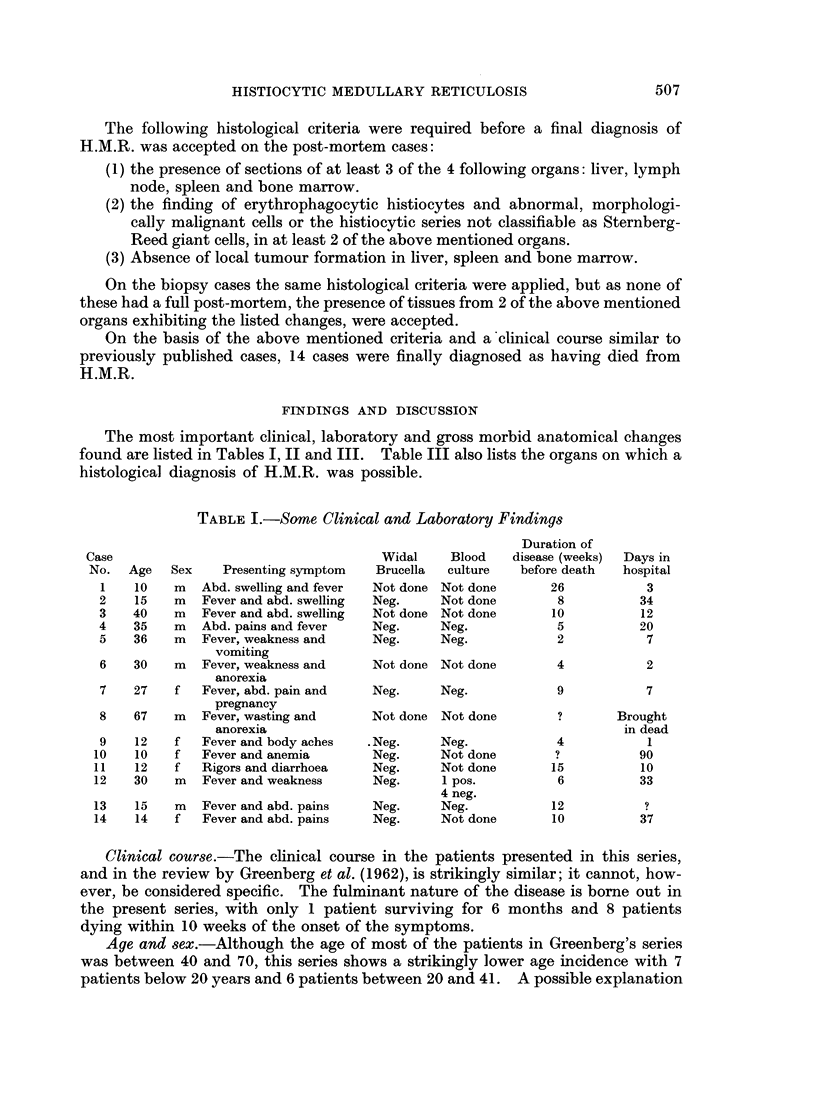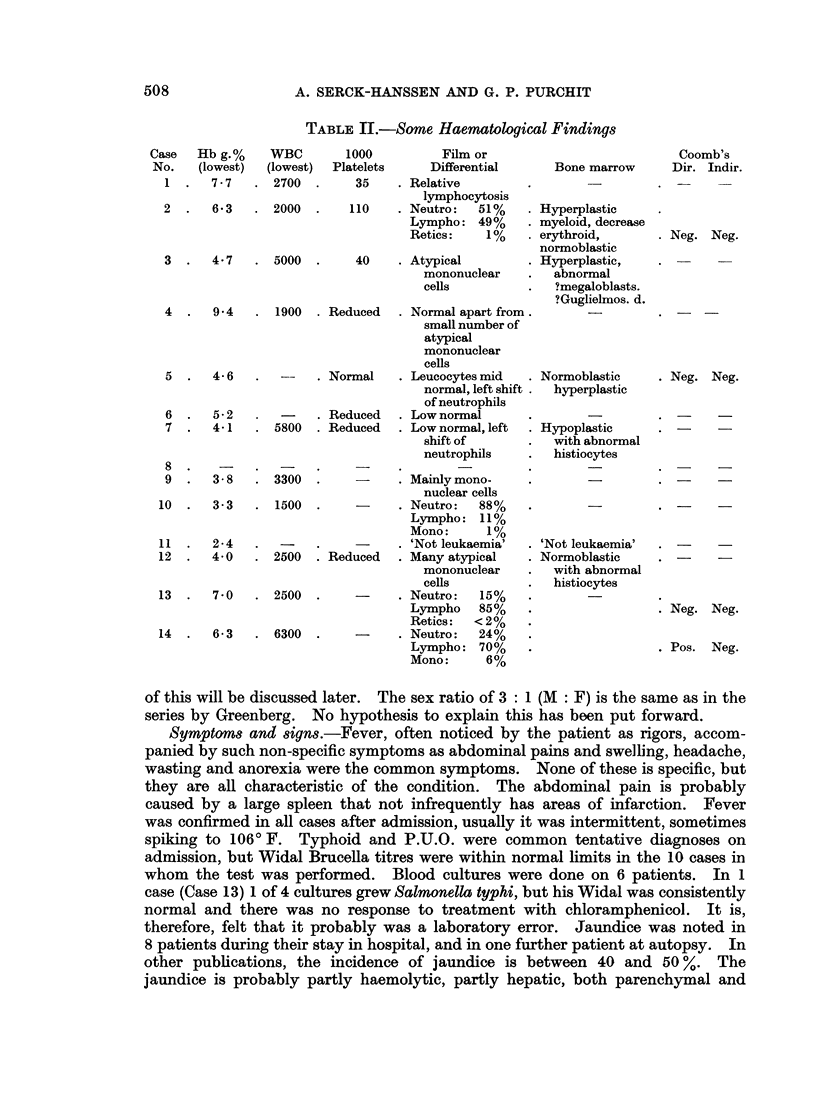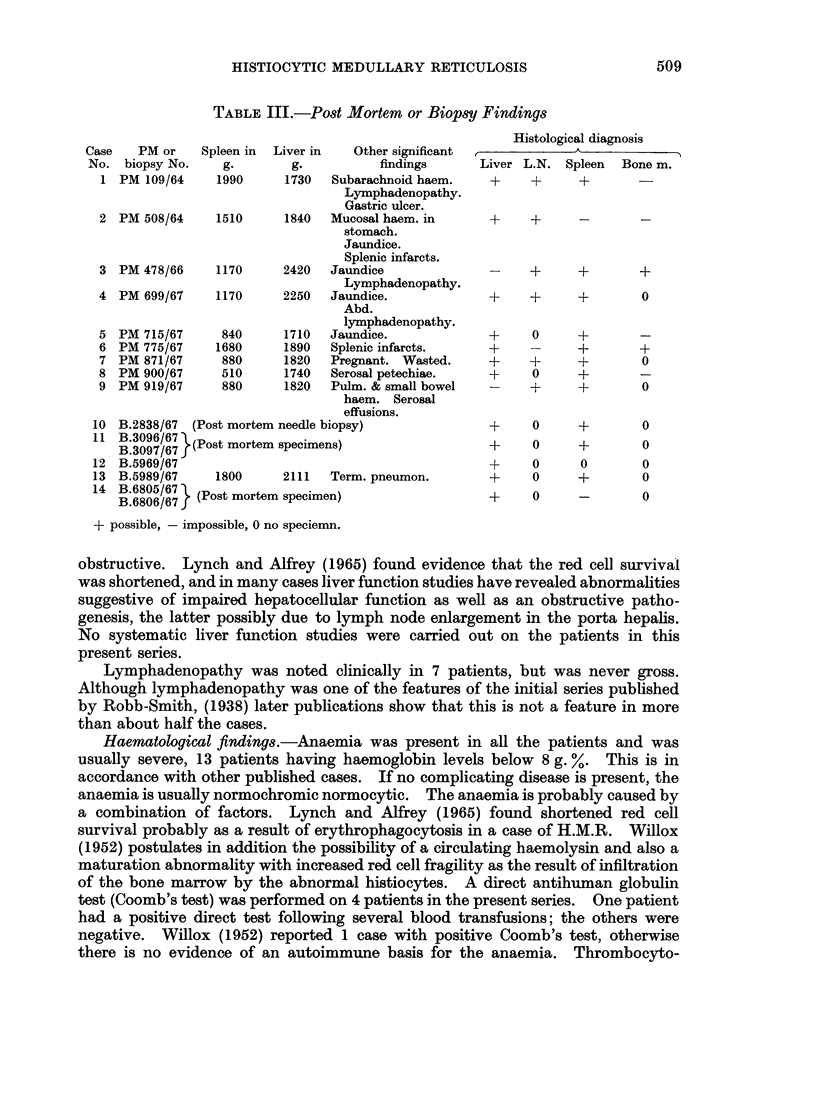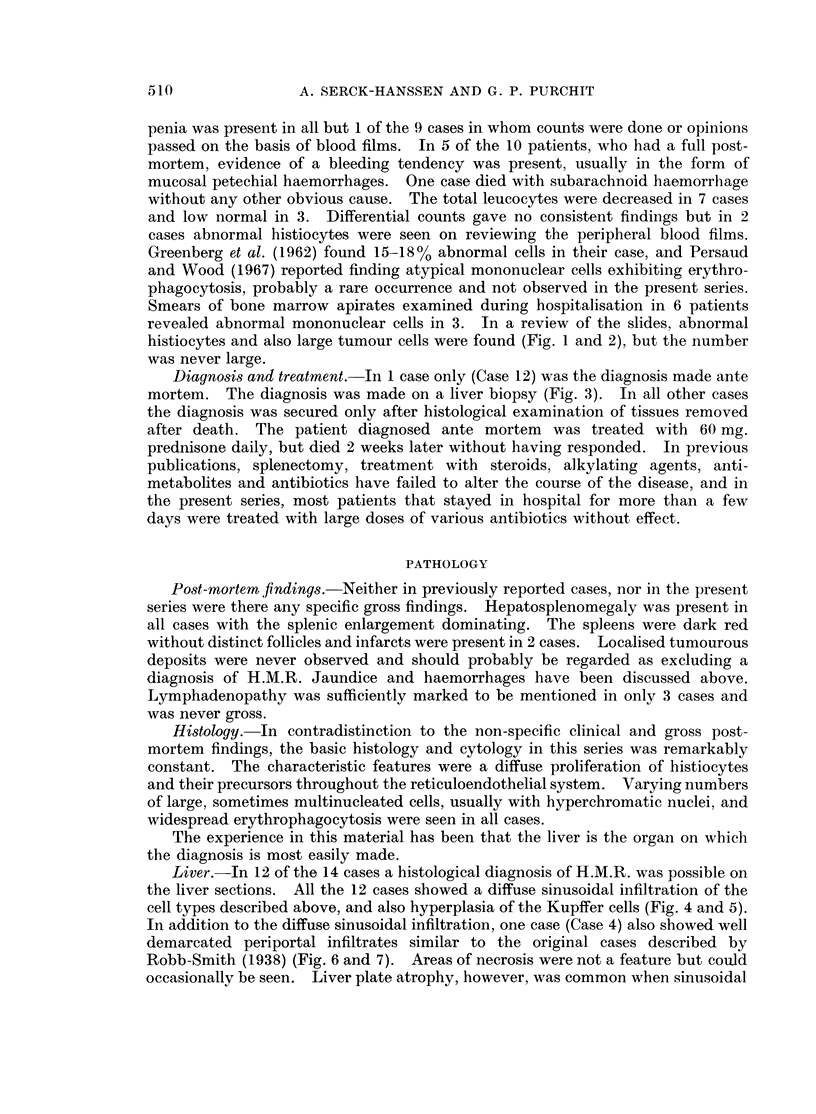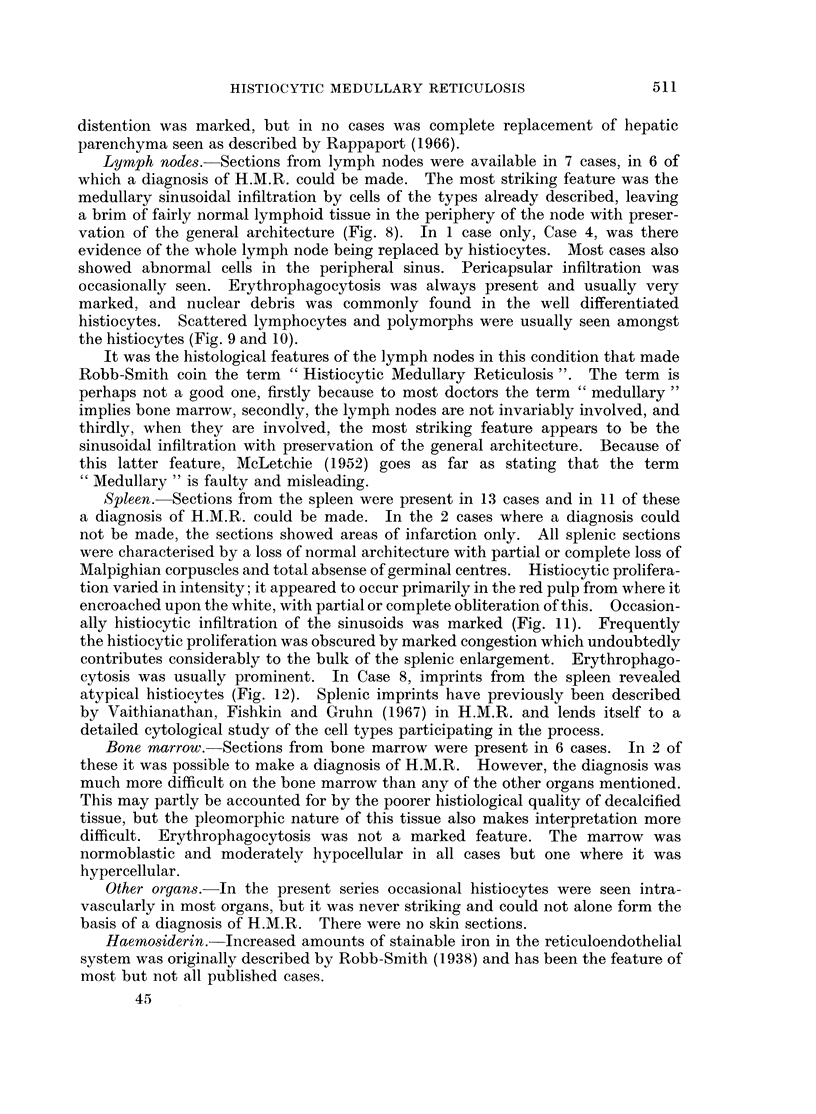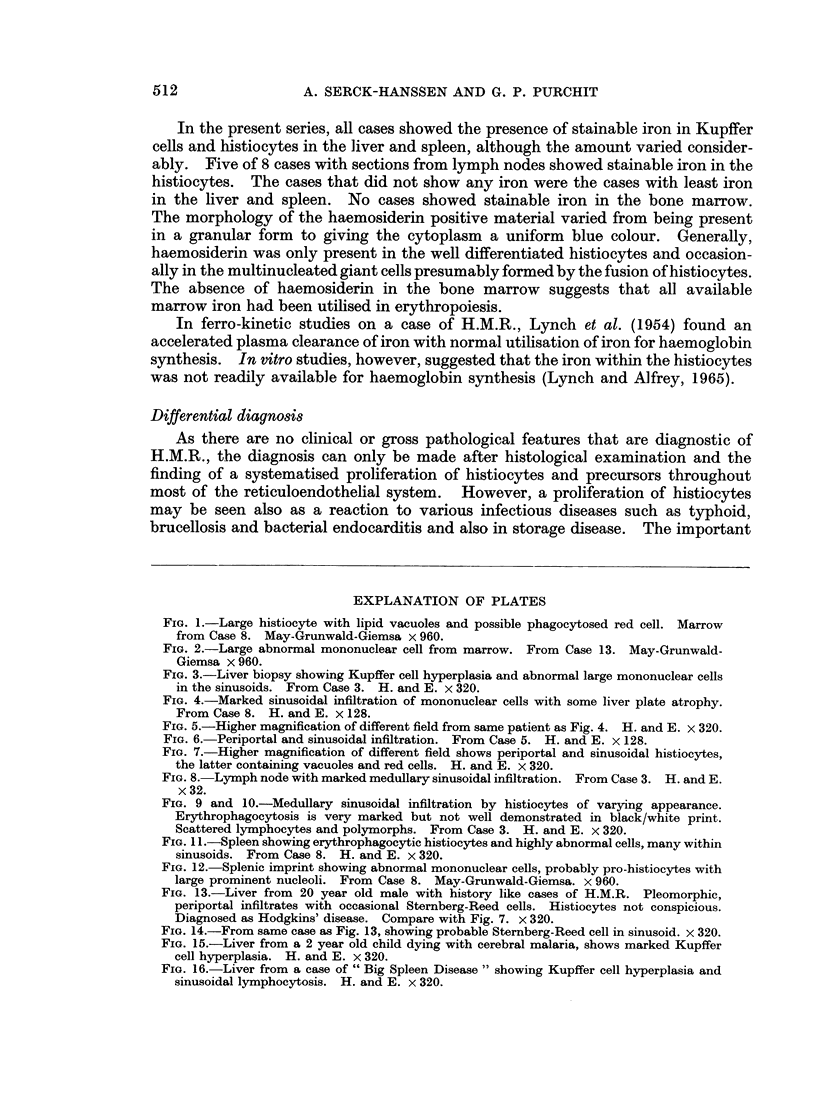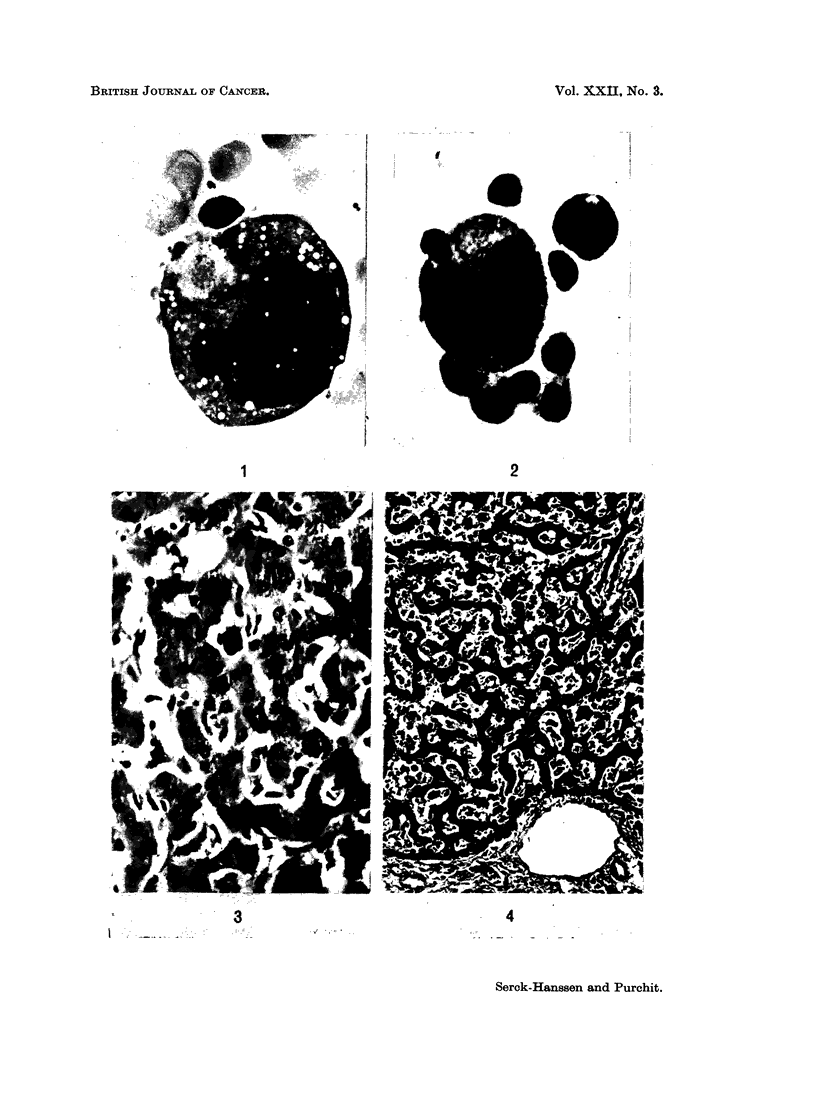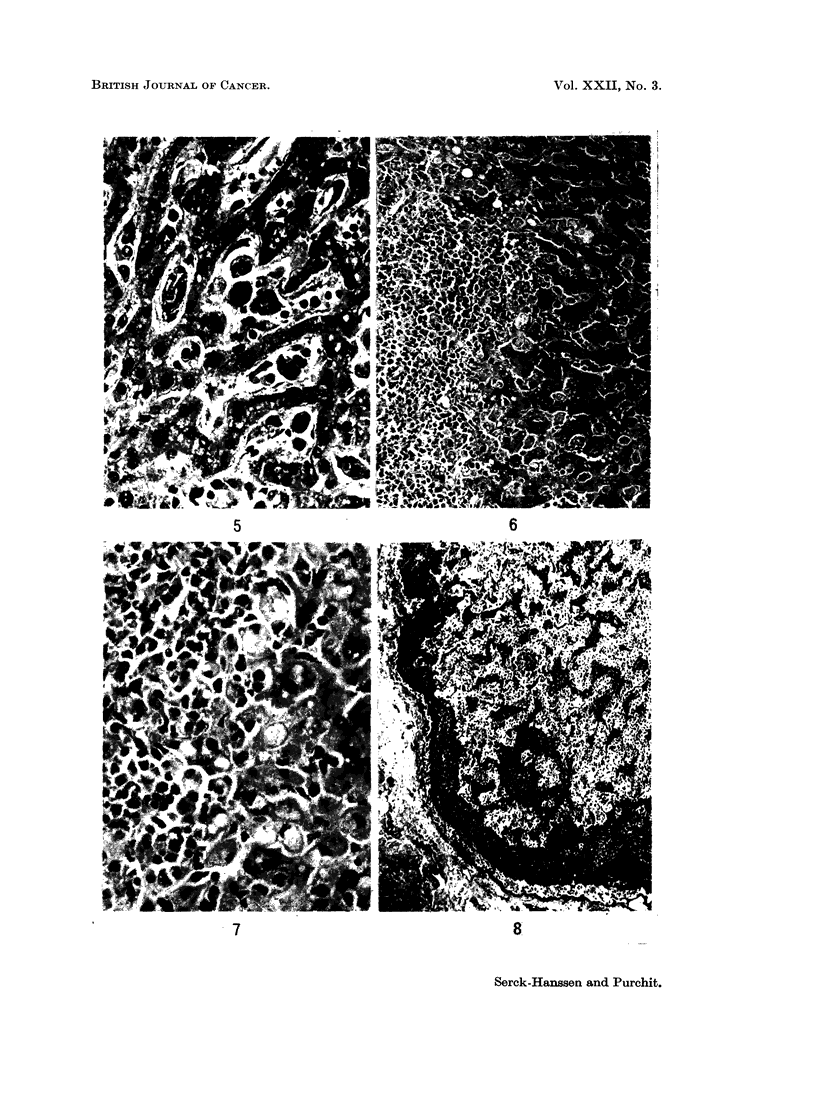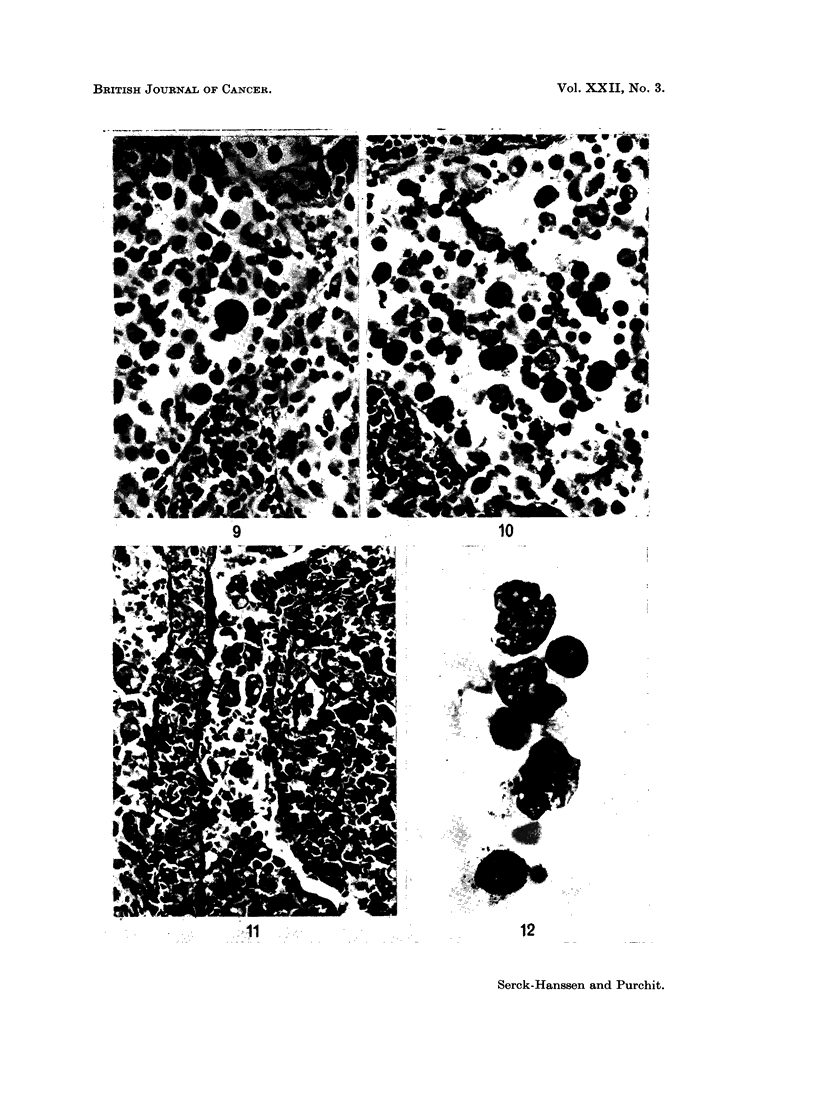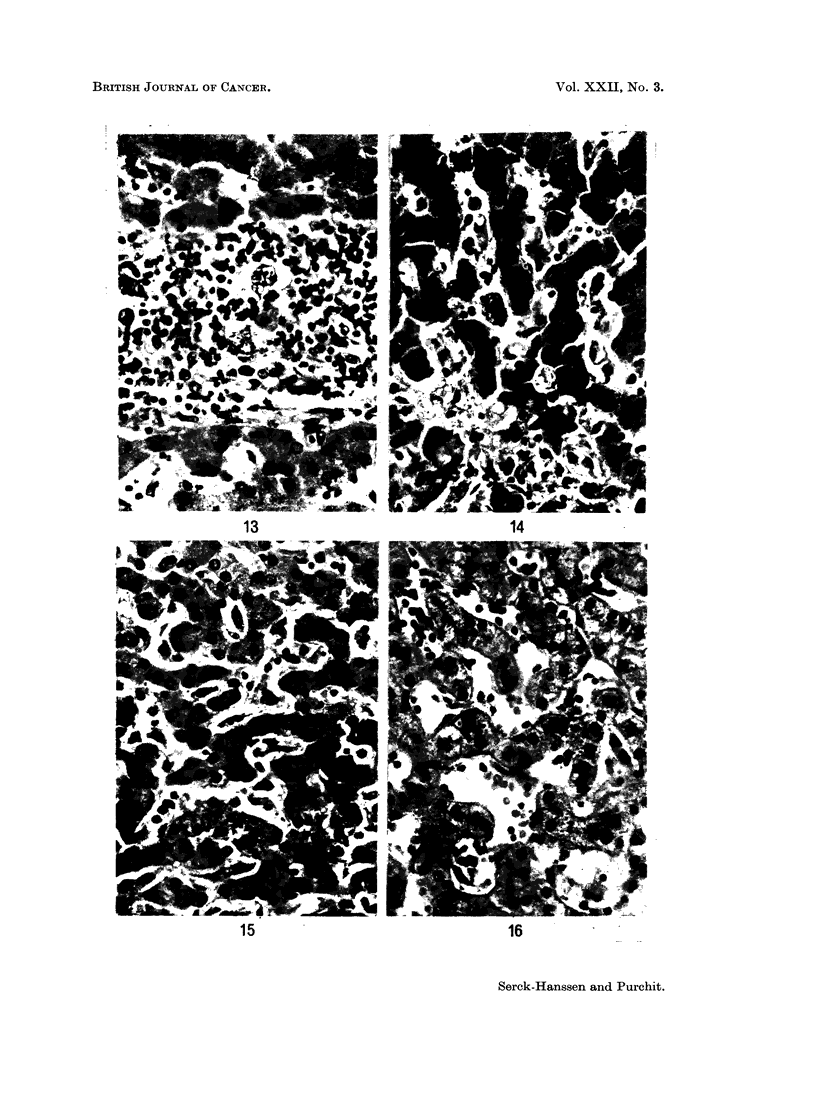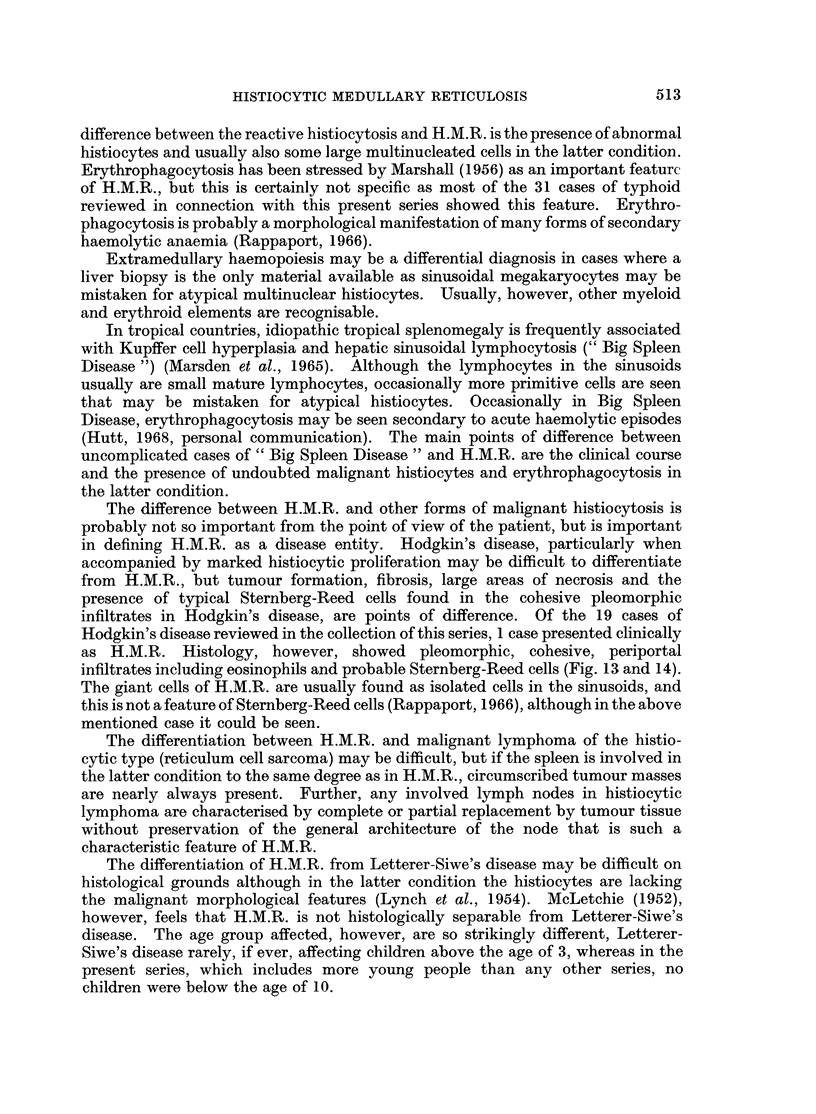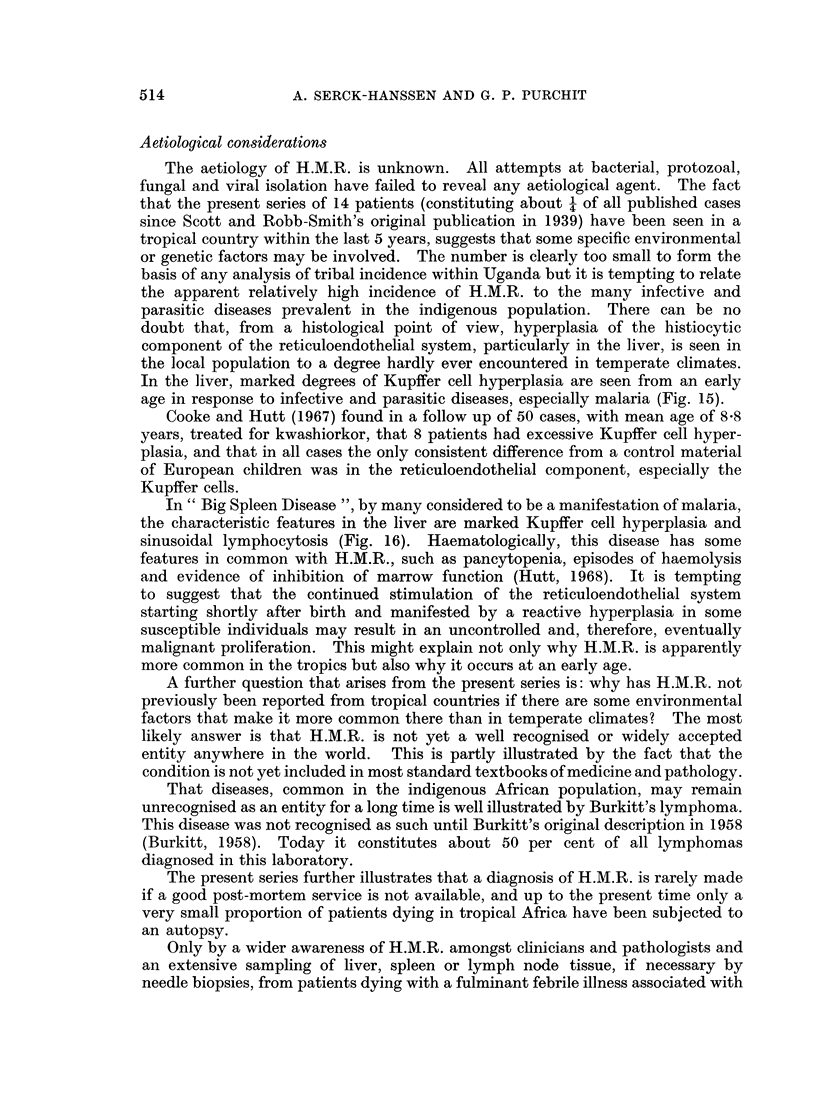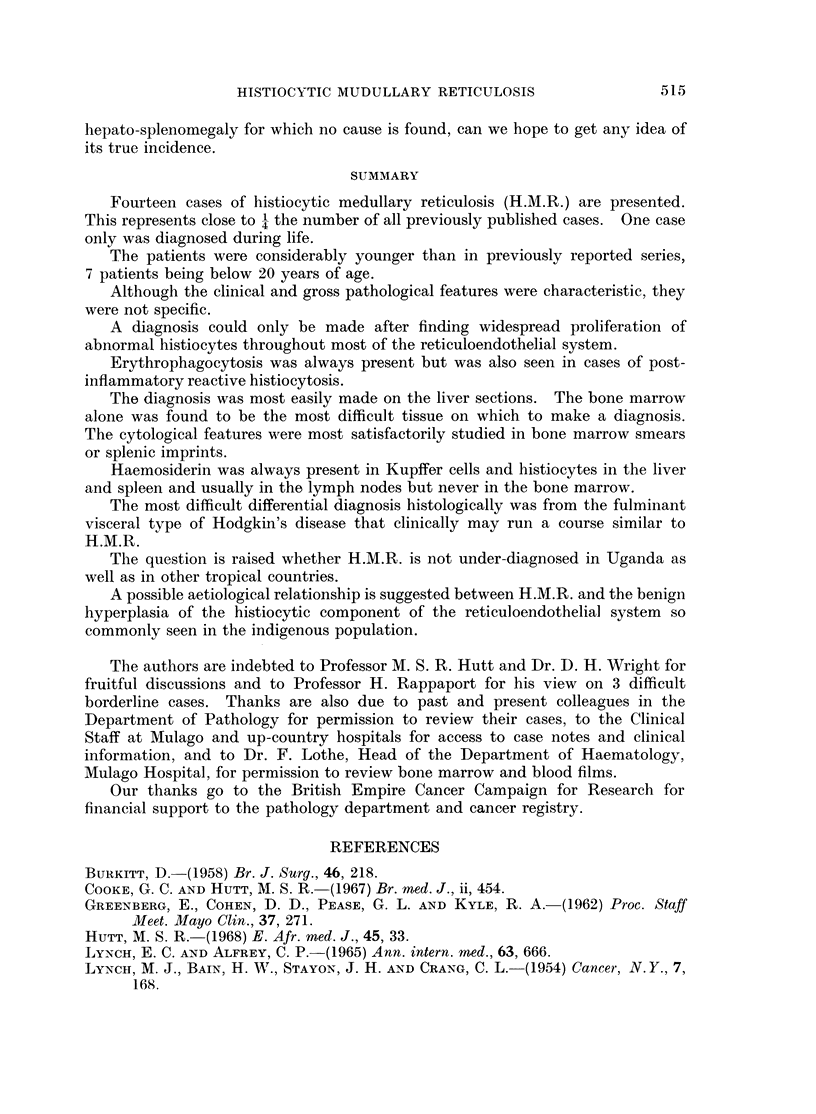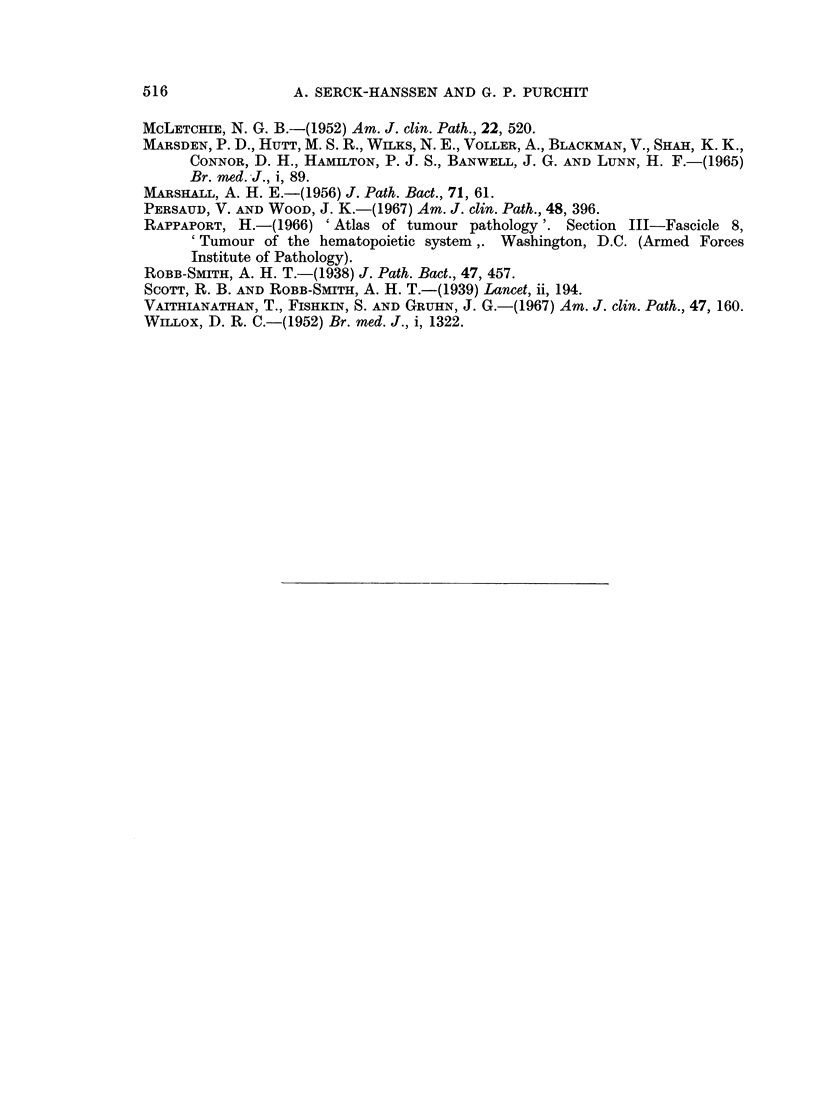# Histiocytic medullary reticulosis. Report of 14 cases from Uganda.

**DOI:** 10.1038/bjc.1968.60

**Published:** 1968-09

**Authors:** A. Serck-Hanssen, G. P. Purchit

## Abstract

**Images:**


### HISTIOCYTIC MEDULLARY RETICULOSIS

### REPORT OF 14 CASES FROM UGANDA

A. SERCK-HANSSEN* AND G. P. PURCHIT†

* *From the Department of Pathology, Makerere University College, Medical School, Kampala, Uganda*

† *Makerere University College, Medical School*

Received for publication May 21, 1968

Histiocytic medullary reticulosis (H.M.R.) was defined as a distinct clinical and pathological entity by Robb-Smith in 1938, and in 1939 Scott and Robb-Smith published a series of 4 personal and 6 collected cases.

The disease, as defined by these authors, is clinically characterised by fever, wasting, generalised lymphadenopathy and hepatosplenomegaly. In the terminal stages, jaundice, purpura, anaemia and leucopenia are common. The disease always ends fatally and the average duration before death is 15 weeks. The pathological findings are a systematised proliferation of erythrophagocytic histiocytes and their precursors throughout the lymphoreticular tissues.

Judging by the number of cases reported in the literature, H.M.R. is a rare disease. According to Persaud and Wood (1967) there were, up to their publication of the disease in a 26 year old Jamaican, 49 reported cases, 25 from Europe, 19 from China, 3 from continental United States and 2 from Hawaii. The largest personal series verified by post-mortems appears to be that of Marshall (1956) who collected 8 cases admitted to the London Hospitals over a period of 25 years.

The purpose of this paper is to report a series of 14 cases from Uganda seen over the last 5 years, 10 of which occurred during 1967.

### MATERIALS AND METHODS

The cases have been collected by going through 4398 records of post-mortems performed at Mulago Hospital during the 5 year period 1963–67, and the 9201 biopsy reports for 1967. All the histological sections on post-mortems with a final diagnosis of 'Big Spleen Disease' (48 cases), thyphoid fever (31 cases), reticuloendotheliosis (21 cases), Hodgkin's disease (19 cases) and H.M.R. (8 cases) were reviewed. On cases that histologically were found to be consistent with, or suggestive of, H.M.R., the clinical records also were reviewed. New sections were cut from the available blocks of liver, spleen, lymph nodes and bone marrow. Sections were stained with haematoxylin and eosin, Turnbull's stain for haemosiderin and selected sections with Gomori's reticulin stain.

Cases from the biopsy register were collected for review when H.M.R. was the suggested histological diagnosis and the clinical course and investigations supported this diagnosis.

* Present address: Department of Pathology, Ulleval Hospital, Oslo, Norway.

The following histological criteria were required before a final diagnosis of H.M.R. was accepted on the post-mortem cases:

(1) the presence of sections of at least 3 of the 4 following organs: liver, lymph node, spleen and bone marrow.
(2) the finding of erythrophagocytic histiocytes and abnormal, morphologically malignant cells or the histiocytic series not classifiable as Sternberg-Reed giant cells, in at least 2 of the above mentioned organs.
(3) Absence of local tumour formation in liver, spleen and bone marrow.

On the biopsy cases the same histological criteria were applied, but as none of these had a full post-mortem, the presence of tissues from 2 of the above mentioned organs exhibiting the listed changes, were accepted.

On the basis of the above mentioned criteria and a clinical course similar to previously published cases, 14 cases were finally diagnosed as having died from H.M.R.

### FINDINGS AND DISCUSSION

The most important clinical, laboratory and gross morbid anatomical changes found are listed in Tables I, II and III. Table III also lists the organs on which a histological diagnosis of H.M.R. was possible.

TABLE I.—*Some Clinical and Laboratory Findings*

| Case No. | Age | Sex | Presenting symptom | Widal Brucella | Blood culture | Duration of disease (weeks) before death | Days in hospital |
|---|---|---|---|---|---|---|---|
| 1 | 10 | m | Abd. swelling and fever | Not done | Not done | 26 | 3 |
| 2 | 15 | m | Fever and abd. swelling | Neg. | Not done | 8 | 34 |
| 3 | 40 | m | Fever and abd. swelling | Not done | Not done | 10 | 12 |
| 4 | 35 | m | Abd. pains and fever | Neg. | Neg. | 5 | 20 |
| 5 | 36 | m | Fever, weakness and vomiting | Neg. | Neg. | 2 | 7 |
| 6 | 30 | m | Fever, weakness and anorexia | Not done | Not done | 4 | 2 |
| 7 | 27 | f | Fever, abd. pain and pregnancy | Neg. | Neg. | 9 | 7 |
| 8 | 67 | m | Fever, wasting and anorexia | Not done | Not done | ? | Brought in dead |
| 9 | 12 | f | Fever and body aches | .Neg. | Neg. | 4 | 1 |
| 10 | 10 | f | Fever and anemia | Neg. | Not done | ? | 90 |
| 11 | 12 | f | Rigors and diarrhoea | Neg. | Not done | 15 | 10 |
| 12 | 30 | m | Fever and weakness | Neg. | 1 pos. 4 neg. | 6 | 33 |
| 13 | 15 | m | Fever and abd. pains | Neg. | Neg. | 12 | ? |
| 14 | 14 | f | Fever and abd. pains | Neg. | Not done | 10 | 37 |

*Clinical course*.—The clinical course in the patients presented in this series, and in the review by Greenberg *et al.* (1962), is strikingly similar; it cannot, however, be considered specific. The fulminant nature of the disease is borne out in the present series, with only 1 patient surviving for 6 months and 8 patients dying within 10 weeks of the onset of the symptoms.

*Age and sex*.—Although the age of most of the patients in Greenberg's series was between 40 and 70, this series shows a strikingly lower age incidence with 7 patients below 20 years and 6 patients between 20 and 41. A possible explanation

TABLE II.—*Some Haematological Findings*

| Case No. | Hb g.% (lowest) | WBC (lowest) | 1000 Platelets | Film or Differential | Bone marrow | Coomb's Dir. | Coomb's Indir. |
|---|---|---|---|---|---|---|---|
| 1 | 7·7 | 2700 | 35 | Relative lymphocytosis | — | — | — |
| 2 | 6·3 | 2000 | 110 | Neutro: 51% Lympho: 49% Retics: 1% | Hyperplastic myeloid, decrease erythroid, normoblastic | Neg. | Neg. |
| 3 | 4·7 | 5000 | 40 | Atypical mononuclear cells | Hyperplastic, abnormal ?megaloblasts. ?Guglielmos. d. | — | — |
| 4 | 9·4 | 1900 | Reduced | Normal apart from small number of atypical mononuclear cells | — | — | — |
| 5 | 4·6 | — | Normal | Leucocytes mid normal, left shift of neutrophils | Normoblastic hyperplastic | Neg. | Neg. |
| 6 | 5·2 | — | Reduced | Low normal | — | — | — |
| 7 | 4·1 | 5800 | Reduced | Low normal, left shift of neutrophils | Hypoplastic with abnormal histiocytes | — | — |
| 8 | — | — | — | — | — | — | — |
| 9 | 3·8 | 3300 | — | Mainly mononuclear cells | — | — | — |
| 10 | 3·3 | 1500 | — | Neutro: 88% Lympho: 11% Mono: 1% | — | — | — |
| 11 | 2·4 | — | — | 'Not leukaemia' | 'Not leukaemia' | — | — |
| 12 | 4·0 | 2500 | Reduced | Many atypical mononuclear cells | Normoblastic with abnormal histiocytes | — | — |
| 13 | 7·0 | 2500 | — | Neutro: 15% Lympho 85% Retics: <2% | — | Neg. | Neg. |
| 14 | 6·3 | 6300 | — | Neutro: 24% Lympho: 70% Mono: 6% | | Pos. | Neg. |

of this will be discussed later. The sex ratio of 3 : 1 (M : F) is the same as in the series by Greenberg. No hypothesis to explain this has been put forward.

*Symptoms and signs.*—Fever, often noticed by the patient as rigors, accompanied by such non-specific symptoms as abdominal pains and swelling, headache, wasting and anorexia were the common symptoms. None of these is specific, but they are all characteristic of the condition. The abdominal pain is probably caused by a large spleen that not infrequently has areas of infarction. Fever was confirmed in all cases after admission, usually it was intermittent, sometimes spiking to 106° F. Typhoid and P.U.O. were common tentative diagnoses on admission, but Widal Brucella titres were within normal limits in the 10 cases in whom the test was performed. Blood cultures were done on 6 patients. In 1 case (Case 13) 1 of 4 cultures grew *Salmonella typhi*, but his Widal was consistently normal and there was no response to treatment with chloramphenicol. It is, therefore, felt that it probably was a laboratory error. Jaundice was noted in 8 patients during their stay in hospital, and in one further patient at autopsy. In other publications, the incidence of jaundice is between 40 and 50%. The jaundice is probably partly haemolytic, partly hepatic, both parenchymal and

TABLE III.—*Post Mortem or Biopsy Findings*

| Case No. | PM or biopsy No. | Spleen in g. | Liver in g. | Other significant findings | Histological diagnosis: Liver | L.N. | Spleen | Bone m. |
|---|---|---|---|---|---|---|---|---|
| 1 | PM 109/64 | 1990 | 1730 | Subarachnoid haem. Lymphadenopathy. Gastric ulcer. | + | + | + | — |
| 2 | PM 508/64 | 1510 | 1840 | Mucosal haem. in stomach. Jaundice. Splenic infarcts. | + | + | — | — |
| 3 | PM 478/66 | 1170 | 2420 | Jaundice Lymphadenopathy. | — | + | + | + |
| 4 | PM 699/67 | 1170 | 2250 | Jaundice. Abd. lymphadenopathy. | + | + | + | 0 |
| 5 | PM 715/67 | 840 | 1710 | Jaundice. | + | 0 | + | — |
| 6 | PM 775/67 | 1680 | 1890 | Splenic infarcts. | + | — | + | + |
| 7 | PM 871/67 | 880 | 1820 | Pregnant. Wasted. | + | + | + | 0 |
| 8 | PM 900/67 | 510 | 1740 | Serosal petechiae. | + | 0 | + | — |
| 9 | PM 919/67 | 880 | 1820 | Pulm. & small bowel haem. Serosal effusions. | — | + | + | 0 |
| 10 | B.2838/67 | (Post mortem needle biopsy) | | | + | 0 | + | 0 |
| 11 | B.3096/67 B.3097/67 | (Post mortem specimens) | | | + | 0 | + | 0 |
| 12 | B.5969/67 | | | | + | 0 | 0 | 0 |
| 13 | B.5989/67 | 1800 | 2111 | Term. pneumon. | + | 0 | + | 0 |
| 14 | B.6805/67 B.6806/67 | (Post mortem specimen) | | | + | 0 | — | 0 |

+ possible, — impossible, 0 no speciemn.

obstructive. Lynch and Alfrey (1965) found evidence that the red cell survival was shortened, and in many cases liver function studies have revealed abnormalities suggestive of impaired hepatocellular function as well as an obstructive pathogenesis, the latter possibly due to lymph node enlargement in the porta hepalis. No systematic liver function studies were carried out on the patients in this present series.

Lymphadenopathy was noted clinically in 7 patients, but was never gross. Although lymphadenopathy was one of the features of the initial series published by Robb-Smith, (1938) later publications show that this is not a feature in more than about half the cases.

*Haematological findings*.—Anaemia was present in all the patients and was usually severe, 13 patients having haemoglobin levels below 8 g. %. This is in accordance with other published cases. If no complicating disease is present, the anaemia is usually normochromic normocytic. The anaemia is probably caused by a combination of factors. Lynch and Alfrey (1965) found shortened red cell survival probably as a result of erythrophagocytosis in a case of H.M.R. Willox (1952) postulates in addition the possibility of a circulating haemolysin and also a maturation abnormality with increased red cell fragility as the result of infiltration of the bone marrow by the abnormal histiocytes. A direct antihuman globulin test (Coomb's test) was performed on 4 patients in the present series. One patient had a positive direct test following several blood transfusions; the others were negative. Willox (1952) reported 1 case with positive Coomb's test, otherwise there is no evidence of an autoimmune basis for the anaemia. Thrombocytopenia was present in all but 1 of the 9 cases in whom counts were done or opinions passed on the basis of blood films. In 5 of the 10 patients, who had a full post-mortem, evidence of a bleeding tendency was present, usually in the form of mucosal petechial haemorrhages. One case died with subarachnoid haemorrhage without any other obvious cause. The total leucocytes were decreased in 7 cases and low normal in 3. Differential counts gave no consistent findings but in 2 cases abnormal histiocytes were seen on reviewing the peripheral blood films. Greenberg *et al.* (1962) found 15–18% abnormal cells in their case, and Persaud and Wood (1967) reported finding atypical mononuclear cells exhibiting erythrophagocytosis, probably a rare occurrence and not observed in the present series. Smears of bone marrow apirates examined during hospitalisation in 6 patients revealed abnormal mononuclear cells in 3. In a review of the slides, abnormal histiocytes and also large tumour cells were found (Fig. 1 and 2), but the number was never large.

*Diagnosis and treatment.*—In 1 case only (Case 12) was the diagnosis made ante mortem. The diagnosis was made on a liver biopsy (Fig. 3). In all other cases the diagnosis was secured only after histological examination of tissues removed after death. The patient diagnosed ante mortem was treated with 60 mg. prednisone daily, but died 2 weeks later without having responded. In previous publications, splenectomy, treatment with steroids, alkylating agents, antimetabolites and antibiotics have failed to alter the course of the disease, and in the present series, most patients that stayed in hospital for more than a few days were treated with large doses of various antibiotics without effect.

### PATHOLOGY

*Post-mortem findings.*—Neither in previously reported cases, nor in the present series were there any specific gross findings. Hepatosplenomegaly was present in all cases with the splenic enlargement dominating. The spleens were dark red without distinct follicles and infarcts were present in 2 cases. Localised tumourous deposits were never observed and should probably be regarded as excluding a diagnosis of H.M.R. Jaundice and haemorrhages have been discussed above. Lymphadenopathy was sufficiently marked to be mentioned in only 3 cases and was never gross.

*Histology.*—In contradistinction to the non-specific clinical and gross post-mortem findings, the basic histology and cytology in this series was remarkably constant. The characteristic features were a diffuse proliferation of histiocytes and their precursors throughout the reticuloendothelial system. Varying numbers of large, sometimes multinucleated cells, usually with hyperchromatic nuclei, and widespread erythrophagocytosis were seen in all cases.

The experience in this material has been that the liver is the organ on which the diagnosis is most easily made.

*Liver.*—In 12 of the 14 cases a histological diagnosis of H.M.R. was possible on the liver sections. All the 12 cases showed a diffuse sinusoidal infiltration of the cell types described above, and also hyperplasia of the Kupffer cells (Fig. 4 and 5). In addition to the diffuse sinusoidal infiltration, one case (Case 4) also showed well demarcated periportal infiltrates similar to the original cases described by Robb-Smith (1938) (Fig. 6 and 7). Areas of necrosis were not a feature but could occasionally be seen. Liver plate atrophy, however, was common when sinusoidal distention was marked, but in no cases was complete replacement of hepatic parenchyma seen as described by Rappaport (1966).

*Lymph nodes.*—Sections from lymph nodes were available in 7 cases, in 6 of which a diagnosis of H.M.R. could be made. The most striking feature was the medullary sinusoidal infiltration by cells of the types already described, leaving a brim of fairly normal lymphoid tissue in the periphery of the node with preservation of the general architecture (Fig. 8). In 1 case only, Case 4, was there evidence of the whole lymph node being replaced by histiocytes. Most cases also showed abnormal cells in the peripheral sinus. Pericapsular infiltration was occasionally seen. Erythrophagocytosis was always present and usually very marked, and nuclear debris was commonly found in the well differentiated histiocytes. Scattered lymphocytes and polymorphs were usually seen amongst the histiocytes (Fig. 9 and 10).

It was the histological features of the lymph nodes in this condition that made Robb-Smith coin the term " Histiocytic Medullary Reticulosis ". The term is perhaps not a good one, firstly because to most doctors the term " medullary " implies bone marrow, secondly, the lymph nodes are not invariably involved, and thirdly, when they are involved, the most striking feature appears to be the sinusoidal infiltration with preservation of the general architecture. Because of this latter feature, McLetchie (1952) goes as far as stating that the term " Medullary " is faulty and misleading.

*Spleen.*—Sections from the spleen were present in 13 cases and in 11 of these a diagnosis of H.M.R. could be made. In the 2 cases where a diagnosis could not be made, the sections showed areas of infarction only. All splenic sections were characterised by a loss of normal architecture with partial or complete loss of Malpighian corpuscles and total absense of germinal centres. Histiocytic proliferation varied in intensity; it appeared to occur primarily in the red pulp from where it encroached upon the white, with partial or complete obliteration of this. Occasionally histiocytic infiltration of the sinusoids was marked (Fig. 11). Frequently the histiocytic proliferation was obscured by marked congestion which undoubtedly contributes considerably to the bulk of the splenic enlargement. Erythrophagocytosis was usually prominent. In Case 8, imprints from the spleen revealed atypical histiocytes (Fig. 12). Splenic imprints have previously been described by Vaithianathan, Fishkin and Gruhn (1967) in H.M.R. and lends itself to a detailed cytological study of the cell types participating in the process.

*Bone marrow.*—Sections from bone marrow were present in 6 cases. In 2 of these it was possible to make a diagnosis of H.M.R. However, the diagnosis was much more difficult on the bone marrow than any of the other organs mentioned. This may partly be accounted for by the poorer histiological quality of decalcified tissue, but the pleomorphic nature of this tissue also makes interpretation more difficult. Erythrophagocytosis was not a marked feature. The marrow was normoblastic and moderately hypocellular in all cases but one where it was hypercellular.

*Other organs.*—In the present series occasional histiocytes were seen intravascularly in most organs, but it was never striking and could not alone form the basis of a diagnosis of H.M.R. There were no skin sections.

*Haemosiderin.*—Increased amounts of stainable iron in the reticuloendothelial system was originally described by Robb-Smith (1938) and has been the feature of most but not all published cases.

In the present series, all cases showed the presence of stainable iron in Kupffer cells and histiocytes in the liver and spleen, although the amount varied considerably. Five of 8 cases with sections from lymph nodes showed stainable iron in the histiocytes. The cases that did not show any iron were the cases with least iron in the liver and spleen. No cases showed stainable iron in the bone marrow. The morphology of the haemosiderin positive material varied from being present in a granular form to giving the cytoplasm a uniform blue colour. Generally, haemosiderin was only present in the well differentiated histiocytes and occasionally in the multinucleated giant cells presumably formed by the fusion of histiocytes. The absence of haemosiderin in the bone marrow suggests that all available marrow iron had been utilised in erythropoiesis.

In ferro-kinetic studies on a case of H.M.R., Lynch *et al.* (1954) found an accelerated plasma clearance of iron with normal utilisation of iron for haemoglobin synthesis. *In vitro* studies, however, suggested that the iron within the histiocytes was not readily available for haemoglobin synthesis (Lynch and Alfrey, 1965).

### *Differential diagnosis*

As there are no clinical or gross pathological features that are diagnostic of H.M.R., the diagnosis can only be made after histological examination and the finding of a systematised proliferation of histiocytes and precursors throughout most of the reticuloendothelial system. However, a proliferation of histiocytes may be seen also as a reaction to various infectious diseases such as typhoid, brucellosis and bacterial endocarditis and also in storage disease. The important

---

### EXPLANATION OF PLATES

FIG. 1.—Large histiocyte with lipid vacuoles and possible phagocytosed red cell. Marrow from Case 8. May-Grunwald-Giemsa × 960.

FIG. 2.—Large abnormal mononuclear cell from marrow. From Case 13. May-Grunwald-Giemsa × 960.

FIG. 3.—Liver biopsy showing Kupffer cell hyperplasia and abnormal large mononuclear cells in the sinusoids. From Case 3. H. and E. × 320.

FIG. 4.—Marked sinusoidal infiltration of mononuclear cells with some liver plate atrophy. From Case 8. H. and E. × 128.

FIG. 5.—Higher magnification of different field from same patient as Fig. 4. H. and E. × 320.

FIG. 6.—Periportal and sinusoidal infiltration. From Case 5. H. and E. × 128.

FIG. 7.—Higher magnification of different field shows periportal and sinusoidal histiocytes, the latter containing vacuoles and red cells. H. and E. × 320.

FIG. 8.—Lymph node with marked medullary sinusoidal infiltration. From Case 3. H. and E. × 32.

FIG. 9 and 10.—Medullary sinusoidal infiltration by histiocytes of varying appearance. Erythrophagocytosis is very marked but not well demonstrated in black/white print. Scattered lymphocytes and polymorphs. From Case 3. H. and E. × 320.

FIG. 11.—Spleen showing erythrophagocytic histiocytes and highly abnormal cells, many within sinusoids. From Case 8. H. and E. × 320.

FIG. 12.—Splenic imprint showing abnormal mononuclear cells, probably pro-histiocytes with large prominent nucleoli. From Case 8. May-Grunwald-Giemsa. × 960.

FIG. 13.—Liver from 20 year old male with history like cases of H.M.R. Pleomorphic, periportal infiltrates with occasional Sternberg-Reed cells. Histiocytes not conspicious. Diagnosed as Hodgkins' disease. Compare with Fig. 7. × 320.

FIG. 14.—From same case as Fig. 13, showing probable Sternberg-Reed cell in sinusoid. × 320.

FIG. 15.—Liver from a 2 year old child dying with cerebral malaria, shows marked Kupffer cell hyperplasia. H. and E. × 320.

FIG. 16.—Liver from a case of " Big Spleen Disease " showing Kupffer cell hyperplasia and sinusoidal lymphocytosis. H. and E. × 320.

1

2

3

4

5

6

7

8

Serck-Hanssen and Purchit.

9 10 11 12

Serck-Hanssen and Purchit.

13

14

15

16

difference between the reactive histiocytosis and H.M.R. is the presence of abnormal histiocytes and usually also some large multinucleated cells in the latter condition. Erythrophagocytosis has been stressed by Marshall (1956) as an important feature of H.M.R., but this is certainly not specific as most of the 31 cases of typhoid reviewed in connection with this present series showed this feature. Erythrophagocytosis is probably a morphological manifestation of many forms of secondary haemolytic anaemia (Rappaport, 1966).

Extramedullary haemopoiesis may be a differential diagnosis in cases where a liver biopsy is the only material available as sinusoidal megakaryocytes may be mistaken for atypical multinuclear histiocytes. Usually, however, other myeloid and erythroid elements are recognisable.

In tropical countries, idiopathic tropical splenomegaly is frequently associated with Kupffer cell hyperplasia and hepatic sinusoidal lymphocytosis (" Big Spleen Disease ") (Marsden *et al.*, 1965). Although the lymphocytes in the sinusoids usually are small mature lymphocytes, occasionally more primitive cells are seen that may be mistaken for atypical histiocytes. Occasionally in Big Spleen Disease, erythrophagocytosis may be seen secondary to acute haemolytic episodes (Hutt, 1968, personal communication). The main points of difference between uncomplicated cases of " Big Spleen Disease " and H.M.R. are the clinical course and the presence of undoubted malignant histiocytes and erythrophagocytosis in the latter condition.

The difference between H.M.R. and other forms of malignant histiocytosis is probably not so important from the point of view of the patient, but is important in defining H.M.R. as a disease entity. Hodgkin's disease, particularly when accompanied by marked histiocytic proliferation may be difficult to differentiate from H.M.R., but tumour formation, fibrosis, large areas of necrosis and the presence of typical Sternberg-Reed cells found in the cohesive pleomorphic infiltrates in Hodgkin's disease, are points of difference. Of the 19 cases of Hodgkin's disease reviewed in the collection of this series, 1 case presented clinically as H.M.R. Histology, however, showed pleomorphic, cohesive, periportal infiltrates including eosinophils and probable Sternberg-Reed cells (Fig. 13 and 14). The giant cells of H.M.R. are usually found as isolated cells in the sinusoids, and this is not a feature of Sternberg-Reed cells (Rappaport, 1966), although in the above mentioned case it could be seen.

The differentiation between H.M.R. and malignant lymphoma of the histiocytic type (reticulum cell sarcoma) may be difficult, but if the spleen is involved in the latter condition to the same degree as in H.M.R., circumscribed tumour masses are nearly always present. Further, any involved lymph nodes in histiocytic lymphoma are characterised by complete or partial replacement by tumour tissue without preservation of the general architecture of the node that is such a characteristic feature of H.M.R.

The differentiation of H.M.R. from Letterer-Siwe's disease may be difficult on histological grounds although in the latter condition the histiocytes are lacking the malignant morphological features (Lynch *et al.*, 1954). McLetchie (1952), however, feels that H.M.R. is not histologically separable from Letterer-Siwe's disease. The age group affected, however, are so strikingly different, Letterer-Siwe's disease rarely, if ever, affecting children above the age of 3, whereas in the present series, which includes more young people than any other series, no children were below the age of 10.

*Aetiological considerations*

The aetiology of H.M.R. is unknown. All attempts at bacterial, protozoal, fungal and viral isolation have failed to reveal any aetiological agent. The fact that the present series of 14 patients (constituting about $\frac{1}{4}$ of all published cases since Scott and Robb-Smith's original publication in 1939) have been seen in a tropical country within the last 5 years, suggests that some specific environmental or genetic factors may be involved. The number is clearly too small to form the basis of any analysis of tribal incidence within Uganda but it is tempting to relate the apparent relatively high incidence of H.M.R. to the many infective and parasitic diseases prevalent in the indigenous population. There can be no doubt that, from a histological point of view, hyperplasia of the histiocytic component of the reticuloendothelial system, particularly in the liver, is seen in the local population to a degree hardly ever encountered in temperate climates. In the liver, marked degrees of Kupffer cell hyperplasia are seen from an early age in response to infective and parasitic diseases, especially malaria (Fig. 15).

Cooke and Hutt (1967) found in a follow up of 50 cases, with mean age of 8·8 years, treated for kwashiorkor, that 8 patients had excessive Kupffer cell hyperplasia, and that in all cases the only consistent difference from a control material of European children was in the reticuloendothelial component, especially the Kupffer cells.

In " Big Spleen Disease ", by many considered to be a manifestation of malaria, the characteristic features in the liver are marked Kupffer cell hyperplasia and sinusoidal lymphocytosis (Fig. 16). Haematologically, this disease has some features in common with H.M.R., such as pancytopenia, episodes of haemolysis and evidence of inhibition of marrow function (Hutt, 1968). It is tempting to suggest that the continued stimulation of the reticuloendothelial system starting shortly after birth and manifested by a reactive hyperplasia in some susceptible individuals may result in an uncontrolled and, therefore, eventually malignant proliferation. This might explain not only why H.M.R. is apparently more common in the tropics but also why it occurs at an early age.

A further question that arises from the present series is: why has H.M.R. not previously been reported from tropical countries if there are some environmental factors that make it more common there than in temperate climates? The most likely answer is that H.M.R. is not yet a well recognised or widely accepted entity anywhere in the world. This is partly illustrated by the fact that the condition is not yet included in most standard textbooks of medicine and pathology.

That diseases, common in the indigenous African population, may remain unrecognised as an entity for a long time is well illustrated by Burkitt's lymphoma. This disease was not recognised as such until Burkitt's original description in 1958 (Burkitt, 1958). Today it constitutes about 50 per cent of all lymphomas diagnosed in this laboratory.

The present series further illustrates that a diagnosis of H.M.R. is rarely made if a good post-mortem service is not available, and up to the present time only a very small proportion of patients dying in tropical Africa have been subjected to an autopsy.

Only by a wider awareness of H.M.R. amongst clinicians and pathologists and an extensive sampling of liver, spleen or lymph node tissue, if necessary by needle biopsies, from patients dying with a fulminant febrile illness associated with hepato-splenomegaly for which no cause is found, can we hope to get any idea of its true incidence.

SUMMARY

Fourteen cases of histiocytic medullary reticulosis (H.M.R.) are presented. This represents close to ¼ the number of all previously published cases. One case only was diagnosed during life.

The patients were considerably younger than in previously reported series, 7 patients being below 20 years of age.

Although the clinical and gross pathological features were characteristic, they were not specific.

A diagnosis could only be made after finding widespread proliferation of abnormal histiocytes throughout most of the reticuloendothelial system.

Erythrophagocytosis was always present but was also seen in cases of post-inflammatory reactive histiocytosis.

The diagnosis was most easily made on the liver sections. The bone marrow alone was found to be the most difficult tissue on which to make a diagnosis. The cytological features were most satisfactorily studied in bone marrow smears or splenic imprints.

Haemosiderin was always present in Kupffer cells and histiocytes in the liver and spleen and usually in the lymph nodes but never in the bone marrow.

The most difficult differential diagnosis histologically was from the fulminant visceral type of Hodgkin's disease that clinically may run a course similar to H.M.R.

The question is raised whether H.M.R. is not under-diagnosed in Uganda as well as in other tropical countries.

A possible aetiological relationship is suggested between H.M.R. and the benign hyperplasia of the histiocytic component of the reticuloendothelial system so commonly seen in the indigenous population.

The authors are indebted to Professor M. S. R. Hutt and Dr. D. H. Wright for fruitful discussions and to Professor H. Rappaport for his view on 3 difficult borderline cases. Thanks are also due to past and present colleagues in the Department of Pathology for permission to review their cases, to the Clinical Staff at Mulago and up-country hospitals for access to case notes and clinical information, and to Dr. F. Lothe, Head of the Department of Haematology, Mulago Hospital, for permission to review bone marrow and blood films.

Our thanks go to the British Empire Cancer Campaign for Research for financial support to the pathology department and cancer registry.

### REFERENCES

Burkitt, D.—(1958) *Br. J. Surg.*, **46**, 218.

Cooke, G. C. and Hutt, M. S. R.—(1967) *Br. med. J.*, ii, 454.

Greenberg, E., Cohen, D. D., Pease, G. L. and Kyle, R. A.—(1962) *Proc. Staff Meet. Mayo Clin.*, **37**, 271.

Hutt, M. S. R.—(1968) *E. Afr. med. J.*, **45**, 33.

Lynch, E. C. and Alfrey, C. P.—(1965) *Ann. intern. med.*, **63**, 666.

Lynch, M. J., Bain, H. W., Stayon, J. H. and Crang, C. L.—(1954) *Cancer, N.Y.*, **7**, 168.

McLetchie, N. G. B.—(1952) *Am. J. clin. Path.*, **22**, 520.
Marsden, P. D., Hutt, M. S. R., Wilks, N. E., Voller, A., Blackman, V., Shah, K. K., Connor, D. H., Hamilton, P. J. S., Banwell, J. G. and Lunn, H. F.—(1965) *Br. med. J.*, i, 89.
Marshall, A. H. E.—(1956) *J. Path. Bact.*, **71**, 61.
Persaud, V. and Wood, J. K.—(1967) *Am. J. clin. Path.*, **48**, 396.
Rappaport, H.—(1966) 'Atlas of tumour pathology'. Section III—Fascicle 8, 'Tumour of the hematopoietic system ,. Washington, D.C. (Armed Forces Institute of Pathology).
Robb-Smith, A. H. T.—(1938) *J. Path. Bact.*, **47**, 457.
Scott, R. B. and Robb-Smith, A. H. T.—(1939) *Lancet*, ii, 194.
Vaithianathan, T., Fishkin, S. and Gruhn, J. G.—(1967) *Am. J. clin. Path.*, **47**, 160.
Willox, D. R. C.—(1952) *Br. med. J.*, i, 1322.