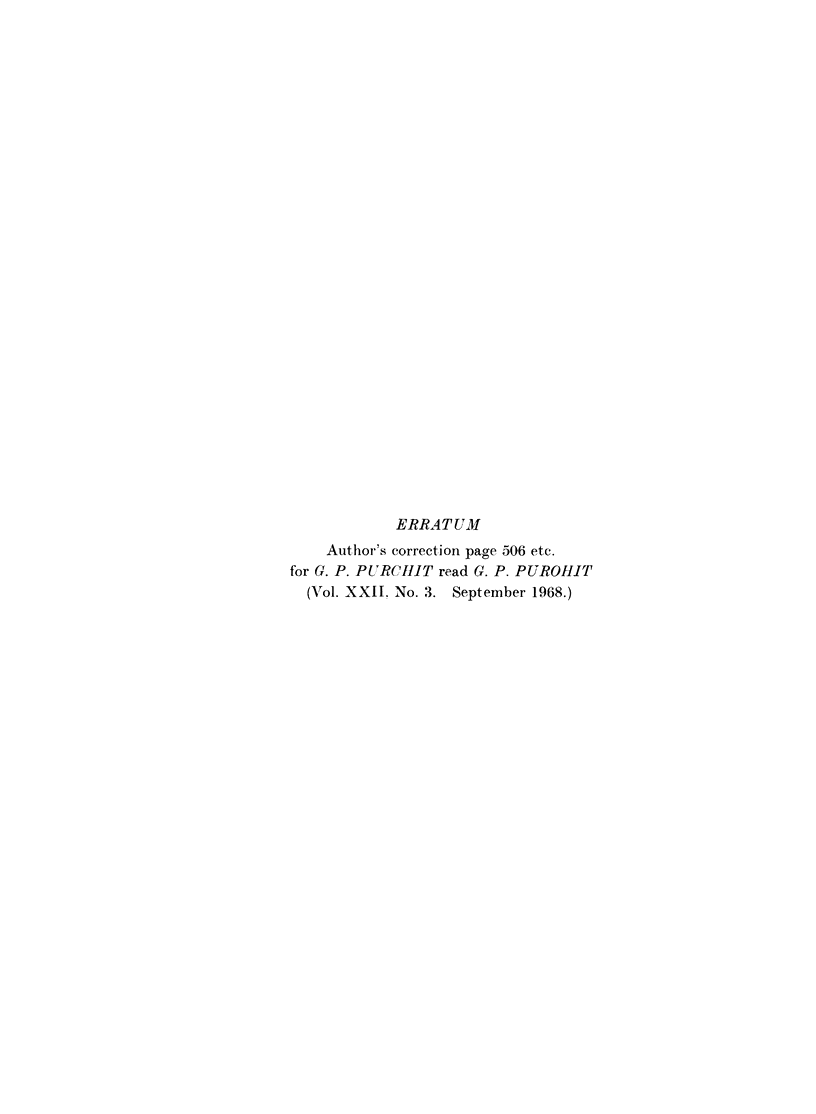# Erratum

**Published:** 1968-12

**Authors:** 


*ERRATUM*

Author's correction page 506 etc.
for *G. P. PURCHIT* read *G. P. PUROHIT*
(Vol. XXII, No. 3. September 1968.)